# A Component Analysis of Endotheliopathy of Trauma

**DOI:** 10.21203/rs.3.rs-9318157/v1

**Published:** 2026-05-19

**Authors:** Chavi Rehani, Sarah Abdullah, Karina Rozenberg, Roumen Vesselinov, Jing-Fei Dong, Rosemary Kozar

**Affiliations:** R Adams Cowley Shock Trauma Center; R Adams Cowley Shock Trauma Center; University of Maryland School of Medicine; University of Maryland; Bloodworks Research Institute; R Adams Cowley Shock Trauma Center

**Keywords:** endotheliopathy, trauma, platelet dysfunction, coagulopathy

## Abstract

Endotheliopathy of trauma (EOT) is a complex response to severe injury involving coagulopathy, platelet dysfunction, endothelial dysfunction, and shock. Although EOT is associated with poor outcomes, the relative contribution of each component is not well defined. This study aimed to determine the impact of individual EOT components on mortality and multiple organ failure (MOF) in severely injured patients. We conducted a single-center prospective observational study from 2022 to 2024 including patients presenting within two hours of injury as a Level 1 trauma activation or with hemorrhage. Demographics, clinical data, and outcomes were collected. Admission blood samples were analyzed for ten biomarkers using a Luminex assay. The primary endpoint was a composite of in-hospital mortality or MOF. Twenty clinical and biomarker variables were grouped into four EOT components. Component-specific multivariable models were developed using ridge regression, and performance was assessed by area under the curve (AUC). Among 159 patients, median age was 39 years, 80% were male, and 42% had penetrating injuries. Median Injury Severity Score was 19, with 15.7% meeting the primary outcome. All component models showed similar predictive performance, highlighting the multifactorial nature of EOT and supporting the need for comprehensive treatment strategies.

## INTRODUCTION

Endotheliopathy of trauma (EOT) is a term that encompasses the complex pathophysiological response to severe injury. It is strongly linked to poor outcomes.^1–3^ EOT was originally characterized by coagulopathy, endothelial dysfunction, and systemic tissue injury, but recent studies have demonstrated that platelet dysfunction may also play a key role.^4–6^ Severe trauma and hemorrhagic shock cause degradation of the endothelial glycocalyx, with shedding of the syndecan-1 ectodomain. Syndecan-1, considered a biomarker for endothelial cell injury, has been strongly correlated with adverse outcomes after trauma. Injured endothelial cells and platelets release inflammatory mediators and other signaling molecules which lead to the dysregulation of platelet plug formation and the clotting cascade, both locally and systemically. Endothelial activation also disrupts the tight and adherens junctions and enhances the permeability of the endothelium. Impairment of the vascular barrier worsens shock physiology and further propagates coagulopathy.^7^ Though the precise mechanisms leading to trauma-induced coagulopathy are incompletely understood, potential contributing factors related to EOT include systemic endothelial injury and platelet dysfunction.^8^ Thus, all components of EOT are interrelated and associated with adverse outcomes.

Blood-based resuscitation with whole blood and blood components, along with goal-directed correction of coagulopathy, form the tenets of management to mitigate EOT and decrease morbidity and mortality in severely injured patients.^9–14^ Though the mechanisms that regulate the individual components of EOT have been studied, the processes that govern the interaction between the components remain poorly defined. Understanding the interactions between individual components of EOT is an essential first step in determining strategies for therapeutic targets. The objective of the current study was to delineate the relative impact of each EOT component on the development of adverse outcomes in severely injured patients.

## METHODS

### Study Design

This study was a secondary analysis of a recently completed prospective observational study of severely injured patients at an urban, high volume, Level 1 Trauma Center. Patients were enrolled between 7/5/2022 and 1/24/2024 at the Shock Trauma Center at the University of Maryland School of Medicine after our Institutional Review Board approval was obtained (HP-00097837). Waiver of consent was granted as admission blood samples were collected from waste blood. Patients met inclusion criteria if they were older than 18 years and presented within 2 hours of injury either as the highest level of trauma activation or with hemorrhage, which was defined as having an initial systolic blood pressure less than 90mmHg attributable to blood loss and requiring blood products (1 or more units of whole blood or 2 or more units of any blood component). Demographics, injury characteristics, vital signs, laboratory values, and outcome measures were abstracted through chart review and use of the trauma registry. The primary composite endpoint was in-hospital mortality or development of multiorgan failure, defined as a Denver multiple organ failure (MOF) score greater than or equal to 3 up to seven days from admission.^15^ Control subjects were age and sex-matched minimally injured patients, defined as having an injury severity score (ISS) less than 9.^16^ All research was performed in accordance with relevant guidelines/regulations and the Declaration of Helsinki.

#### Luminex Assay for Protein Quantification

Blood samples were collected from all study patients upon arrival. Plasma was isolated by centrifugation at 5000rpm for 10 minutes and frozen at −80C. The levels of plasma biomarkers for platelet dysfunction, endothelial dysfunction, and/or coagulation were determined using a fluorescent bead-based multiplexed immunoassay (Luminex Assay, R&D systems). Plasma was incubated with fluorescent antibodies conjugated to magnetic beads for 2 hours at room temperature, and antibody binding was quantified on a Luminex 200 machine from Luminex xMAP technology per the manufacturer instructions. The data were analyzed using Luminex xPONENT software. The measured analytes included interleukin-6 (IL-6) as an indicator of inflammation associated with endothelial cell dysfunction, angiopoietin-1 (ANG1), angiopoietin-2 (ANG2), syndecan-1, von Willebrand factor A2 domain (vWF), and a disintegrin and metalloprotease with thrombospondin type I motif member 13 (ADAMTS13) as indicators of endothelial injury/activation,^17–20^ P-selectin, tissue factor, and vWF as measures of platelet dysfunction,^21, 22^ and tissue factor, vWF, ADAMTS13, thrombomodulin, and D-dimer as measures of coagulopathy.^19, 20, 23, 24^

#### Multivariable Modeling and Statistical Analysis

Twenty variables, including all measured analytes and select clinical data (lactate, fibrinogen, prothrombin time (PT), partial thromboplastin time (PTT), lowest ED systolic blood pressure (SBP), and shock index), were grouped into one or more of the four EOT components. Multivariable models were then generated for each EOT component (Table 1). A model combining all components was also generated using all available variables. Because some variables within each model were likely to be highly correlated, the ridge regression regularization technique was used to minimize the effects of multicollinearity. Model performance was assessed using the Area Under the Receiver Operating Characteristic Curve (AUC). Data is presented as median [interquartile range] and AUC (95% confidence interval).

## RESULTS

159 patients met inclusion criteria for severe injury. The median age was 39 years [31–53], 80% were male, and 42% sustained penetrating trauma. The median Injury Severity Score (ISS) was 19 [11–27] and 15.7% of patients met the primary composite outcome. In the age- and sex-matched control group of minimally injured patients (n = 39), the median age was 37 years [28–54], 79% were male, and the median ISS was 1 [1–2] (Table 2).

**Figure 1:**
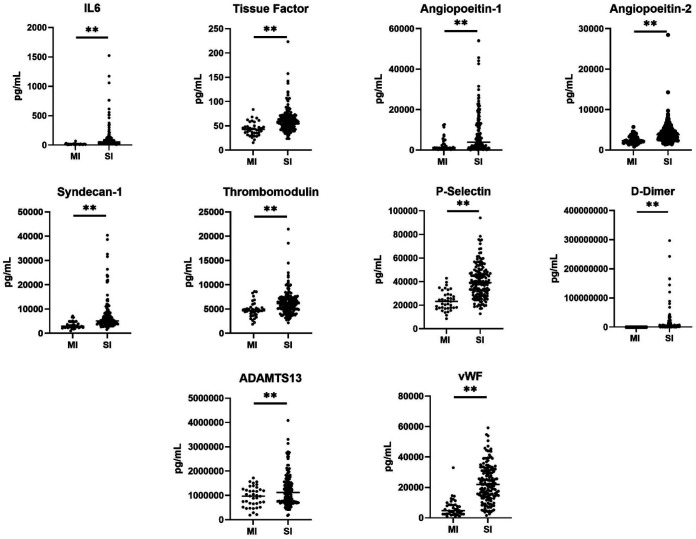
Comparing Analyte Concentrations of Minimally and Severely Injured Patients: The dot plot for each analyte illustrates the difference in concentration (pg/mL) between minimally (MI) and severely (SI) injured patients. Statistically significant differences (p < 0.001) are annotated by **.

**Figure 2:**
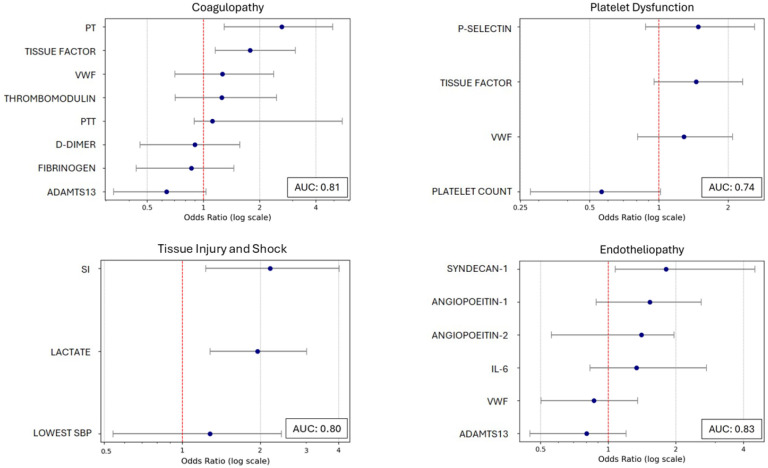
Component-Specific Models of Endotheliopathy of Trauma in Severely Injured Patients: The graph for each component illustrates the effect of its included variables on the primary outcome of mortality or multiorgan failure in severely injured patients. The area under the curve (AUC) of each model is shown in the bottom right inset of its respective graph. The odds ratio shown for each variable is relative to the other variables included in that model.

**Figure 3:**
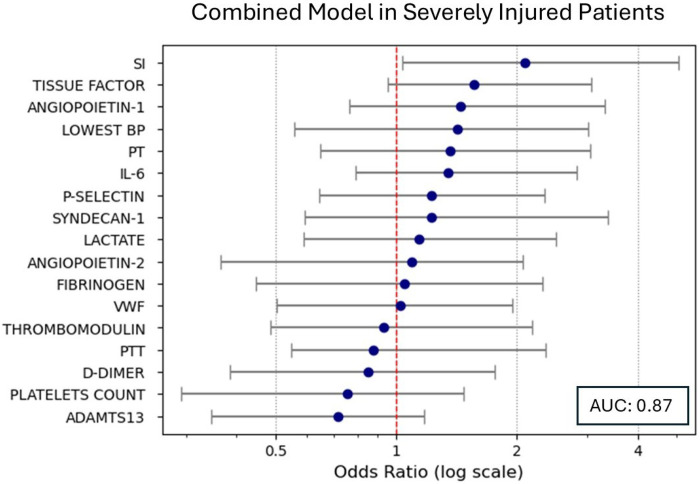
Combined Model of Endotheliopathy of Trauma in Severely Injured Patients: The graph illustrates the effect of all included variables on the primary outcome of mortality or multiorgan failure. The area under the curve (AUC) is shown in the bottom right inset.

**Figure 4:**
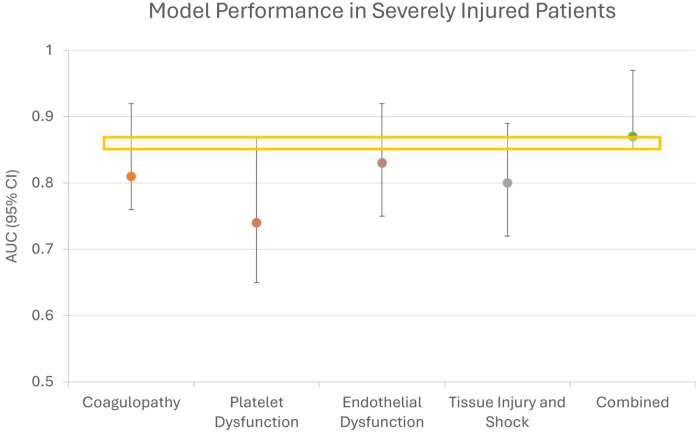
Model AUCs and 95% Confidence Intervals: The graph shows the area under the curve (AUC) and the respective 95% confidence intervals for all models. The range of overlapping intervals is highlighted.

When compared to minimally injured controls, severely injured patients had statistically significant increases in the plasma concentrations of all measured analytes, consistent with systemic inflammation, endothelial dysfunction, platelet abnormalities, and shock (Fig. 1). The AUCs of component-specific models were: coagulopathy 0.81 (0.76–0.92), platelet dysfunction 0.74 (0.65–0.87), endothelial dysfunction 0.83 (0.75–0.92), and shock 0.80 (0.72–0.89) (Fig. 2). The combined model using all variables had an AUC of 0.87 (0.85–0.97) (Fig. 3). The predictive performance of each component-specific model was not only statistically comparable to that of every other component model, but also to that of the combined model (Fig. 4).

## DISCUSSION

Through this prospective observational study, we aimed to gain insight into the complex interplay between the various pathological processes that contribute to the development of EOT. The components included in this analysis were coagulopathy, platelet dysfunction, endothelial dysfunction, and shock. Though the traditional understanding of EOT did not include platelet dysfunction, mounting evidence suggests that dysregulated platelets are significant contributors to EOT via the release of inflammatory mediators that regulate thrombosis and vascular permeability.^5, 6^ Our results support the findings of these studies, in that, like the other components, platelet dysfunction as a component of EOT was also significantly associated with adverse outcomes. In fact, all studied components of EOT were statistically comparable predictors of in-hospital mortality or multiorgan failure in severely injured patients. Our findings suggest that each component of the EOT similarly and significantly contributes to adverse outcomes after trauma.

To date, efforts to address the EOT and improve outcomes have focused on blood-based resuscitation.^14^ Multiple landmark trials have demonstrated that early resuscitation with fresh frozen plasma improves outcomes in patients with hemorrhagic shock.^10–12^ Today, the early use of plasma is replacing crystalloid resuscitation, not only for patients with hemorrhagic shock but also those with sepsis and burn injuries due to its endothelial protective effects.^25–27^ Clinical and preclinical evidence suggest that the protective effects of plasma are due, in part, to maintenance of the glycocalyx.^28^ Plasma administration has been shown to be associated with a decrease in endothelial and glycocalyx biomarkers such as syndecan-1.^29^ Both cryoprecipitate and prothrombin concentrates have also been shown to have endothelial protective effects.^30, 31^ Recently, whole blood has re-emerged as a promising resuscitative option for prehospital and in-hospital use, based on a growing body of evidence showing improved outcomes in military and civilian settings.^32, 33^ Whole blood has demonstrated benefits in targeting the endothelial glycocalyx and vascular permeability. In a rat model of hemorrhagic shock, whole blood and FFP demonstrated similar protective effects, both restoring endothelial glycocalyx thickness to sham levels and reducing microvascular permeability.^34, 35^ Platelets have been another focus of multiple recent studies, highlighting previously underappreciated protective and immunomodulatory effects.^36–38^ Platelets are key regulators of the vascular endothelium, but they appear to have diverging roles under varying pathologic conditions. In animal models of inflammatory states, platelets have been shown to increase vascular permeability.^39, 40^ However, *in vitro* and *in vivo* models of hemorrhagic shock and trauma show that platelet transfusion may mitigate vascular leak.^41^ Gallagher et al. showed that releasates found in activated platelets from healthy patients may mitigate endotheliopathy in trauma patients by disrupting redox reactions and increasing antioxidants.^38^ Other studies have found that higher platelet-to-red blood cell transfusion ratios are associated with improved outcomes in severely injured patients with hemorrhagic shock, and earlier administration of those platelets is associated with protective effects.^10, 42, 43^ However, despite these and other significant efforts to improve outcomes with advanced resuscitation strategies that target a component of the EOT, patients are still succumbing to both hemorrhage and multiple organ failure. Additional therapeutics are needed to further improve outcomes.

The results of this study highlight the need for therapeutics across all four components of EOT to improve morbidity and mortality in severely injured patients. Because all components were found to contribute similarly to outcomes, it follows that potential therapeutics that function across more than one component may be the most effective targets. Among the analytes measured in this study, those that have the most overlap across components were tissue factor, vWF, and ADAMTS13. In fact, there is growing evidence that addressing the imbalance of the vWF-ADAMTS13 axis can mitigate EOT and improve outcomes, including the use of human recombinant ADAMTS13 in a preclinical model of trauma.^44–46^ A number of strategies have also been investigated to regulate the tissue factor pathway such as recombinant thrombomodulin (rTM) and an anti-tissue factor pathway inhibitor monoclonal antibody (marstacimab), but studies have largely been limited to patients with sepsis and hemophilia, respectively.^47, 48^

This study has several limitations. Data was collected from a single center and included a small sample size. Furthermore, because this work is a secondary analysis of a recently completed prospective observational trial, the variables available for use in this analysis were predetermined. This led to differences in the number of variables that were included in each model, which likely contributed to models with fewer variables having wider AUC confidence intervals. Thromboelastography (TEG) and arterial blood gas data was not collected for every patient, which prohibited the use of base deficit and additional coagulation and platelet variables that may have strengthened the models. Additional biomarkers may have also strengthened the models. Lastly, mechanism of injury (blunt vs. penetrating) and patient sex (female vs. male) have both been shown to affect mortality risk and immune response to injury.^49–51^ In this study, the limited number of patients with adverse outcomes in the female and penetrating injury groups precluded subanalyses to determine differences between the sexes and mechanisms of injury. Future prospective studies with a larger sample size would help to mitigate this challenge.

In conclusion, this study demonstrates that coagulopathy, platelet dysfunction, endothelial dysfunction, and shock contribute comparably and significantly to adverse outcomes following severe injury. Our results underscore the individual importance of each component and the multifactorial nature of EOT. These findings support a paradigm in which effective therapeutics focus on variables that span multiple components of EOT and on developing targeted component-based therapies to improve outcomes in critically injured patients.

## Figures and Tables

**Table 1:** Endotheliopathy of Trauma Component-Specific Multivariable Models and Included Variables

Coagulopathy	Platelet Dysfunction	Endothelial Dysfunction	Shock
Prothrombin Time (PT)	P-selectin	Interleukin-6 (IL-6)	Lactate
Partial Thromboplastin Time (PTT)	Tissue Factor	Syndecan-1	Lowest SBP
Tissue Factor	von Willebrand Factor (vWF)	Angiopoeitin-1 (ANG1)	Shock Index
von Willebrand Factor (vWF)	Platelet Count	Angiopoeitin-2 (ANG2)	
ADAMTS13		von Willebrand Factor (vWF)	
Thrombomodulin		ADAMTS13	
D-dimer			
Fibrinogen			

SBP = systolic blood pressure

**Table 2:** Study Patient Demographics

*Age (years)*	Minimally Injured (n = 39)	Severely Injured (n = 159)
	37 [28–54]	39 [31–54]
*Sex, Male (%)*	79	80
*Penetrating Trauma (%)*	57.9	42.1*
*ISS*	1 [1–2]	19 [11–27]*
*Mortality or MOF (%)*	0	15.7*

Median [interquartile range]

* p < 0.05 vs. minimally injured controls

## Data Availability

The datasets generated during and/or analyzed during the current study are available from the corresponding author on reasonable request.

## References

[1] Naumann D. N. Endotheliopathy of Trauma is an on-Scene Phenomenon, and is Associated with Multiple Organ Dysfunction Syndrome: A Prospective Observational Study. Shock 49, 420–428 (2018). 10.1097/shk.0000000000000999 28945676

[2] Johansson P. I. Traumatic Endotheliopathy: A Prospective Observational Study of 424 Severely Injured Patients. Ann. Surg. 265, 597–603. 10.1097/sla.0000000000001751 (2017). 27144442 PMC5300027

[3] Teeter W. Trauma-Induced Coagulopathy: Prevalence and Association with Mortality Persist 20 Years Later. Shock 62, 380–385 (2024). 10.1097/shk.0000000000002416 38920139

[4] Jenkins D. H. Trauma hemostasis and oxygenation research position paper on remote damage control resuscitation: definitions, current practice, and knowledge gaps. Shock 41 Suppl 1, 3–12. 10.1097/shk.0000000000000140 (2014). PMC4309265 24430539

[5] Kutcher M. E. Characterization of platelet dysfunction after trauma. J. Trauma. Acute Care Surg. 73, 13–19. 10.1097/TA.0b013e318256deab (2012). 22743367 PMC3387387

[6] Vulliamy P. Alterations in platelet behavior after major trauma: adaptive or maladaptive? Platelets 32, 295–304 (2021). 10.1080/09537104.2020.1718633 31986948 PMC7382983

[7] Nair A. B., Schreiber M. A. & Pati S. in *in* Trauma Induced Coagulopathy. 117–133 (eds Hunter B., Moore M. D., Neal, Ernest E. & Moore) (Springer International Publishing, 2021).

[8] Buzzard L. & Schreiber M. Trauma-induced coagulopathy: What you need to know. J. Trauma. Acute Care Surg. 96, 179–185. 10.1097/ta.0000000000004170 (2024). 37828662

[9] Holcomb J. B. The prospective, observational, multicenter, major trauma transfusion (PROMMTT) study: comparative effectiveness of a time-varying treatment with competing risks. JAMA Surg. 148, 127–136. 10.1001/2013.jamasurg.387 (2013). 23560283 PMC3740072

[10] Holcomb J. B. Transfusion of plasma, platelets, and red blood cells in a 1:1:1 vs a 1:1:2 ratio and mortality in patients with severe trauma: the PROPPR randomized clinical trial. Jama 313, 471–482. 10.1001/jama.2015.12 (2015). 25647203 PMC4374744

[11] Sperry J. L. Prehospital Plasma during Air Medical Transport in Trauma Patients at Risk for Hemorrhagic Shock. N Engl. J. Med. 379, 315–326. 10.1056/NEJMoa1802345 (2018). 30044935

[12] Pati S. Protective effects of fresh frozen plasma on vascular endothelial permeability, coagulation, and resuscitation after hemorrhagic shock are time dependent and diminish between days 0 and 5 after thaw. J. Trauma. 69 (Suppl 1), S55–63. 10.1097/TA.0b013e3181e453d4 (2010). 20622621 PMC3126659

[13] Wu F. Resuscitative Strategies to Modulate the Endotheliopathy of Trauma: From Cell to Patient. Shock 53, 575–584. 10.1097/shk.0000000000001378 (2020). 31090680 PMC6842415

[14] Cardenas J. C., Dong J. F. & Kozar R. A. Injury-induced endotheliopathy: What you need to know. J. Trauma. Acute Care Surg. 95, 454–463. 10.1097/ta.0000000000004082 (2023). 37314417 PMC10527751

[15] Sauaia A. Temporal trends of postinjury multiple-organ failure: still resource intensive, morbid, and lethal. J. Trauma. Acute Care Surg. 76, 582–592. 10.1097/ta.0000000000000147 (2014). discussion 592 – 583. 24553523 PMC4116088

[16] Zeineddin A. Biomarkers of endothelial cell dysfunction persist beyond resuscitation in patients with hemorrhagic shock. J. Trauma. Acute Care Surg. 93, 572–578. 10.1097/ta.0000000000003758 (2022). 35939376 PMC9613546

[17] Yu W. K. Angiopoietin-2 outperforms other endothelial biomarkers associated with severe acute kidney injury in patients with severe sepsis and respiratory failure. Crit. Care. 25, 48. 10.1186/s13054-021-03474-z (2021). 33541396 PMC7859898

[18] Gonzalez Rodriguez E. Syndecan-1: A Quantitative Marker for the Endotheliopathy of Trauma. J. Am. Coll. Surg. 225, 419–427. 10.1016/j.jamcollsurg.2017.05.012 (2017). 28579548

[19] Chauhan A. K. The combined roles of ADAMTS13 and VWF in murine models of TTP, endotoxemia, and thrombosis. Blood 111, 3452–3457. 10.1182/blood-2007-08-108571 (2008). 18083848 PMC2275014

[20] Chen J., Chung D. W. & Inflammation von Willebrand factor, and ADAMTS13. Blood 132, 141–147 (2018). 10.1182/blood-2018-02-769000 29866815 PMC6043979

[21] Ferroni P. Biomarkers of platelet activation in acute coronary syndromes. Thromb. Haemost. 108, 1109–1123. 10.1160/TH12-08-0550 (2012). 23014768

[22] Ludwig R. J., Schön M. P. & Boehncke W. H. P-selectin. Expert Opin. Ther. Targets. 11, 1103–1117. 10.1517/14728222.11.8.1103 (2007). 17665981

[23] Wada H. Plasma thrombomodulin as a marker of vascular disorders in thrombotic thrombocytopenic purpura and disseminated intravascular coagulation. Am. J. Hematol. 39, 20–24. 10.1002/ajh.2830390106 (1992). 1311143

[24] Watanabe-Kusunoki K., Nakazawa D., Ishizu A. & Atsumi T. Thrombomodulin as a Physiological Modulator of Intravascular Injury. Front. Immunol. 11, 575890. 10.3389/fimmu.2020.575890 (2020). 33042158 PMC7525002

[25] van den Brink D. P. Plasma as a resuscitation fluid for volume-depleted shock: Potential benefits and risks. Transfusion 61 (Suppl 1), S301–s312. 10.1111/trf.16462 (2021). 34057210 PMC8361764

[26] Cartotto R. & Callum J. A. Review on the Use of Plasma During Acute Burn Resuscitation. J. Burn Care Res. 41, 433–440. 10.1093/jbcr/irz184 (2020). 31734693

[27] Holcomb J. B. & Pati S. Optimal trauma resuscitation with plasma as the primary resuscitative fluid: the surgeon’s perspective. Hematology Am Soc Hematol Educ Program 656–659 (2013). (2013). 10.1182/asheducation-2013.1.656 24319247

[28] Chipman A. M. Fresh frozen plasma attenuates lung injury in a novel model of prolonged hypotensive resuscitation. J. Trauma. Acute Care Surg. 89, S118–s125. 10.1097/ta.0000000000002719 (2020). 32282752 PMC7830779

[29] Gruen D. S. Prehospital tranexamic acid is associated with a dose-dependent decrease in syndecan-1 after trauma: A secondary analysis of a prospective randomized trial. J. Trauma. Acute Care Surg. 95, 642–648. 10.1097/ta.0000000000003955 (2023). 37125811 PMC10615664

[30] Barry M. Cryoprecipitate attenuates the endotheliopathy of trauma in mice subjected to hemorrhagic shock and trauma. J. Trauma. Acute Care Surg. 90, 1022–1031. 10.1097/ta.0000000000003164 (2021). 33797484 PMC8141010

[31] Zeineddin A. Early lyophilized cryoprecipitate enhances the ADAMTS13/VWF ratio to reduce systemic endotheliopathy and lessen lung injury in a mouse multiple-trauma hemorrhage model. J. Trauma. Acute Care Surg. 95, S137–s143. 10.1097/ta.0000000000004065 (2023). 37211640 PMC10389395

[32] Spinella P. C., Perkins J. G., Grathwohl K. W., Beekley A. C. & Holcomb J. B. Warm fresh whole blood is independently associated with improved survival for patients with combat-related traumatic injuries. J. Trauma. 66, S69–76. 10.1097/TA.0b013e31819d85fb (2009). 19359973 PMC3126655

[33] Jones A. R. & Frazier S. K. Increased mortality in adult patients with trauma transfused with blood components compared with whole blood. J. Trauma. Nurs. 21, 22–29. 10.1097/jtn.0000000000000025 (2014). 24399315 PMC4126240

[34] Torres Filho I. P., Torres L. N., Salgado C. & Dubick M. A. Plasma syndecan-1 and heparan sulfate correlate with microvascular glycocalyx degradation in hemorrhaged rats after different resuscitation fluids. Am. J. Physiol. Heart Circ. Physiol. 310, H1468–1478. 10.1152/ajpheart.00006.2016 (2016). 27037369

[35] Torres L. N., Sondeen J. L. & Dubick M. A. Torres Filho, I. Systemic and microvascular effects of resuscitation with blood products after severe hemorrhage in rats. J. Trauma. Acute Care Surg. 77, 716–723. 10.1097/ta.0000000000000448 (2014). 25494423

[36] Barrett T. J. Platelets amplify endotheliopathy in COVID-19. Sci. Adv. 7, eabh2434. 10.1126/sciadv.abh2434 (2021). 34516880 PMC8442885

[37] Fields A. T. A new trauma frontier: Exploratory pilot study of platelet transcriptomics in trauma patients. J. Trauma. Acute Care Surg. 92, 313–322. 10.1097/ta.0000000000003450 (2022). 34738997 PMC8781218

[38] Gallagher L. T. Platelet releasates mitigate the endotheliopathy of trauma. J. Trauma. Acute Care Surg. 97, 738–746. 10.1097/ta.0000000000004342 (2024). 38764145 PMC11502277

[39] Rayes J., Bourne J. H., Brill A. & Watson S. P. The dual role of platelet-innate immune cell interactions in thrombo-inflammation. Res. Pract. Thromb. Haemost. 4, 23–35. 10.1002/rth2.12266 (2020). 31989082 PMC6971330

[40] Yang X. The Crucial Roles of Platelets as Immune Mediators in Sepsis. J. Inflamm. Res. 18, 12825–12845. 10.2147/jir.S535701 (2025). 40979493 PMC12449891

[41] Trivedi A. Freeze-dried platelets promote clot formation, attenuate endothelial cell permeability, and decrease pulmonary vascular leak in a murine model of hemorrhagic shock. J. Trauma. Acute Care Surg. 90, 203–214. 10.1097/ta.0000000000002984 (2021). 33060537

[42] Holcomb J. B. Increased platelet:RBC ratios are associated with improved survival after massive transfusion. J. Trauma. 71, 318–328. 10.1097/TA.0b013e318227edbb (2011). 21814099

[43] Cardenas J. C. Platelet transfusions improve hemostasis and survival in a substudy of the prospective, randomized PROPPR trial. Blood Adv. 2, 1696–1704. 10.1182/bloodadvances.2018017699 (2018). 30030268 PMC6058234

[44] Fogarty H. Sustained VWF-ADAMTS-13 axis imbalance and endotheliopathy in long COVID syndrome is related to immune dysfunction. J. Thromb. Haemost. 20, 2429–2438. 10.1111/jth.15830 (2022). 35875995 PMC9349977

[45] Gao D. Recombinant ADAMTS-13 Improves Survival of Mice Subjected to Endotoxemia. Int. J. Mol. Sci. 24 10.3390/ijms241411782 (2023). PMC10380474 37511541

[46] Kleinveld D. J. B. Plasma and rhADAMTS13 reduce trauma-induced organ failure by restoring the ADAMTS13-VWF axis. Blood Adv. 5, 3478–3491. 10.1182/bloodadvances.2021004404 (2021). 34505883 PMC8525227

[47] Yamakawa K., Murao S. & Aihara M. Recombinant Human Soluble Thrombomodulin in Sepsis-Induced Coagulopathy: An Updated Systematic Review and Meta-Analysis. Thromb. Haemost. 119, 56–65. 10.1055/s-0038-1676345 (2019). 30597500

[48] Matino D. Marstacimab prophylaxis in hemophilia A/B without inhibitors: results from the phase 3 BASIS trial. Blood 146, 1654–1663. 10.1182/blood.2024027468 (2025). 40608864 PMC12824696

[49] Bösch F., Angele M. K. & Chaudry I. H. Gender differences in trauma, shock and sepsis. Mil Med. Res. 5, 35. 10.1186/s40779-018-0182-5 (2018). 30360757 PMC6203206

[50] Coleman J. R. Sex dimorphisms in coagulation: Implications in trauma-induced coagulopathy and trauma resuscitation. Am. J. Hematol. 99 (Suppl 1), S28–s35. 10.1002/ajh.27296 (2024). 38567625 PMC11380117

[51] Donohue J. K. Mechanism matters: mortality and endothelial cell damage marker differences between blunt and penetrating traumatic injuries across three prehospital clinical trials. Sci. Rep. 14, 2747. 10.1038/s41598-024-53398-1 (2024). 38302619 PMC10834504

